# Effects of COVID-19 on Urban Population Flow in China

**DOI:** 10.3390/ijerph18041617

**Published:** 2021-02-08

**Authors:** Xiaorong Jiang, Wei Wei, Shenglan Wang, Tao Zhang, Chengpeng Lu

**Affiliations:** 1 College of Resource Environment and Tourism, Hubei University of Arts and Science, Xiangyang 441053, China; jiangxr@hbuas.edu.cn (X.J.); weiwei@xy.hbuas.edu.cn (W.W.); wangshenglan05@163.com (S.W.); 2 Institute of County Economic Development & Rural Revitalization Strategy, Lanzhou University, Lanzhou 730000, China

**Keywords:** urban population flow, complex networks analysis, effective distance model, COVID-19, China

## Abstract

The COVID-19 epidemic has become a Public Health Emergency of International Concern. Thus, this sudden health incident has brought great risk and pressure to the city with dense population flow. A deep understanding of the migration characteristics and laws of the urban population in China will play a very positive role in the prevention and control of the epidemic situation. Based on Baidu location-based service (LBS) big data, using complex networks method and geographic visualization tools, this paper explores the spatial structure evolution of population flow network (PFN) in 368 cities of China under different traffic control situations. Effective distance models and linear regression models were established to analyze how the population flow across cities affects the spread of the epidemic. Our findings show that: (1) the scope of population flow is closely related to the administrative level of the city and the traffic control policies in various cities which adjust with the epidemic situation; The PFN mainly presents the hierarchical structure dominated by the urban hierarchy and the regional isolation structure adjacent to the geographical location.(2) through the analysis network topology structure of PFN, it is found that only the first stage has a large clustering coefficient and a relatively short average path length, which conforms to the characteristics of small world network. The epidemic situation has a great impact on the network topology in other stages, and the network structure tends to be centralized. (3) The overall migration scale of the whole country decreased by 36.85% compared with the same period of last year’s lunar calendar, and a further reduction of 78.52% in the nationwide traffic control stage after the festival. (4) Finally, based on the comparison of the effective distance and the spatial distance from the Wuhan to other destination cities, it is demonstrated that there is a higher correlation between the effective distance and the epidemic spread both in Hubei province and the whole country.

## 1. Introduction

During the Spring Festival of 2020, the epidemic of COVID-19 broke out in Wuhan, Hubei Province, and spread rapidly to the whole country. All cities have taken strict traffic control measures, such as blockading cities, closing down high-speed, civil aviation and railways, restrictions on travel, strict home and other policies, resulting in the city’s external traffic disruption, enterprise shutdown, etc. This not only brings challenges to urban management, but also has a profound impact on the daily life of urban residents [1,2]. After the cities in Hubei Province were closed on 23 January 2020, the provinces in China launched the response to major public health emergencies (level-1). The strictest and most thorough traffic control measures quickly and effectively curb the spread of the epidemic momentum. However, at the beginning of the Spring Festival Travel Rush (started in 10 January 2020), a large number of people concentrated in returning home, visiting relatives and returning to the city during this period [3], these mobile populations contain a large number of unrecorded infections, which appears to have facilitated the rapid spread of the virus throughout China [4]. In the past, we just had to face the pressure of severe shortage of transportation infrastructure supply. However, this superimposed epidemic situation will inevitably trigger a chain reaction to the nationwide population flow. China’s special population mobility is also a key reason for the increased risk of the epidemic. Therefore, a scientific understanding of the temporal and spatial network characteristics of urban population flow in China under the background of epidemic situation and Spring Festival transportation is of great significance for formulating effective prevention and control measures and reflecting on urban population management policies.

Population flow is one of the largest and most far-reaching geographical processes in China since its reform and opening [5]. It is a social phenomenon as well as a geographical one [3]. Population flow will continue to be an important driver of urbanization [6]. The large-scale population flow has a great impact on the transportation network system, industrial development and social culture. The positioning and scale of urban functions will also change accordingly [7,8]. Under the present information background, the network big data has become the important information carrier which records the resident social activity path. Compared with traditional census data, these massive spatiotemporal data have the natural advantages of large sample size, multi-source data and strong analysis and prediction, and have become increasingly important data sources for urban space and human behavior pattern research [9,10,11]. The human behavior big data (e.g., mobile check-in data, cellular signaling data, taxi trajectory data, bus card swiping records, social network check-in data, and bank card swiping records) have been widely used in sensing the geographical environment [12], assisting sustainable economic development [13], and recognizing urban structure and functional areas [14]. In particular, population migration between the source city and the destination city, as a social expression of the spatial interaction, can intuitively reflect the spatial patterns of urban network [15,16,17].

At the same time, the risk of the pandemic in megacities because of the population aggregation has received attention [18,19,20]. Population migration, a key role in the transmission of diseases, often lead to outbreaks of acute diseases and the spread of infectious diseases in specific regions [21,22,23]. Therefore, tracking population flows is especially important in the context of the outbreak of COVID-19 [24], many scholars at home and abroad analyzed the spread characteristics, factors affecting virus transmission and prevention and control measures of COVID-19 from the angle of population flow and traffic control. Chinazzi et al. (2020) used global air travel data and the global epidemic and mobility model (GLEAM) to simulate and predict the global transmission path and visualization of a novel pneumonia epidemic based on a multi-scenario scenario [25]. Using mobile phone location data. Jia et al. (2020) and Badr et al. (2020) found a high correlation between the number of infections and mobility in each prefecture/county in China and the United States [24,26]. A retrospective simulation model by Kraemer et al. (2020) showed that the lockdown did, to a large extent, limit the spread of the virus [2]. Tian et al. (2020) found that the closure of Wuhan city reduced the speed of the epidemic spreading to other cities by 2.91 days (95% CI: 2.54–3.29 days), thus delaying the spread of the disease in other regions of China [27]. Based on the data of population flow and the epidemic situation of new coronavirus in Wuhan, Zhou et al. (2020) preliminarily calculated the exact number of people from Wuhan to other places during the Spring Festival, and suggested that special attention should be paid to rural areas in the prevention and control of the epidemic [28]. Liu Yong et al. (2020) evaluated the spatial and temporal characteristics of COVID-19 epidemic and the risk of population movement based on the data of 1243 cases in Henan Province in China [29].

While the existing studies have revealed the positive correlation between human mobility and transmissions, the dynamics of the relationship between these two are not yet quantified, especially the impact of traffic blockade policy on population travel during the epidemic period. In addition, due to the fact that the COVID-19 outbreak in Chinese Spring Festival, the total population migration changed rapidly and dynamically. Geographic heterogeneity of epidemic transmission cannot be ignored [30]. Therefore, it is necessary to develop a universal model of population mobility based on a geographical network perspective that is suitable for different stages and spatial scales. Based on the migration data of Baidu LBS among 368 cities in China from 1 January to 23 March in 2020, this paper uses the complex network method to study how COVID-19 drives spatio-temporal evolution of population flow network (PFN) in China. The followed contents organized as follows: Section 2 focusing on the complex network analysis method and explains the selection of data and indicators; Section 3 studies the spatial characteristics and evolution of PFN at different stages of traffic control; and then Section 4 is to present the dynamics of the relationship analysis result between population flow and transmissions. Finally, the conclusion and discussion will be drawn out in the fifth part.

## 2. Materials and Methods 

### 2.1. Data Sources

The population flow data in the study were sourced from Baidu Qianxi (migration) data provided by the open LBS platform of Baidu (http://qianxi.baidu.om/). According to the adjustment of the government’s traffic control policy, we divide the research period into five stages. The lockdown of Wuhan City on 23 January 2020 was taken as the dividing line, and the first stage is from 1 January to 23 January, a large floating population flows from work cities to their home cities before the Spring Festival, forming the “return migration stage”; The second stage is the Spring Festival holiday, from 24 January to 2 February (the Spring Festival holiday has been extended for 3 days due to the epidemic), the scale of population flow dropped sharply under the dual influence of epidemic control and holidays break; February 9 is the first day of the government’s plan to resume production. until then, people’s travel is strictly controlled; Obviously, the long-term stagnation of population flow is not conducive to the national epidemic prevention and control work and the convenience of people’s daily life. Some enterprises which related to epidemic prevention and control and people’s livelihood take the lead in resuming production; Until 26 February, a total of 21 provinces lowered the emergency response to level 2, and began to lift relevant travel restrictions in an orderly manner in succession. All over the country, the population migration is in an orderly state of recovery. 

Baidu Qianxi data covers 368 cities in China, including municipalities, cities (autonomous prefectures, leagues), provincial cities and some county administrative units in Hainan Province. Baidu migration index (BMI), which is the result of indexation of the actual number of migrants, is used to indicate the population flow scale between cities in Baidu Qianxi data. As the days of each period are different, the average daily BMI is used as the numerical calculation. The data on confirmed cases of COVID-19 is mainly from the daily report on the COVID-19 outbreak published on the official websites of the National Health Commission (NHC) and the health commissions of 31 provinces, autonomous regions and municipalities in China. The COVID-19 cumulative number of confirmed cases by province and city until 23 March 2020. 

### 2.2. Research Framework and Methods

#### 2.2.1. Overall Methodological Framework

**Figure 1:**
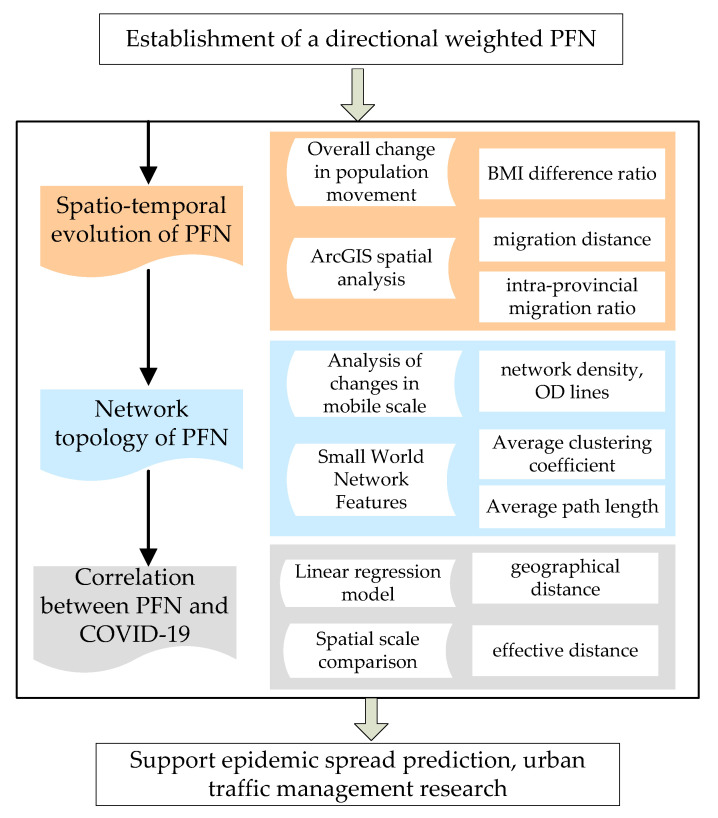
The methodological framework of the geospatial network analysis. PFN: population flow network.

This paper proposes a geospatial network analytical framework to analyze the effects of COVID-19 on PFN from the perspectives of GIS and complex network science. Three major steps are taken to complete the analysis, as shown in Figure 1. Firstly, the Spatial and temporal evolution of PFN is explored based on the total BMI difference ratio between 2020 and 2019, migration distance and intra-provincial migration ratio calculated by ArcGIS spatial analysis tool. Secondly, network density, average path length and clustering coefficient are applied to further observe the network structure characteristics of PFN in China in five stages. Finally, effective distance model is used to reveal the correlation of COVID-19 transmission with PFN. The methods are described in detail in the following sections.

#### 2.2.2. Social Network Analysis

Using the social network analysis method, we established a 368 × 68 directed weighting matrix, in which taking BMI between cities as the weight. This paper uses 3 network indicators, including the network density, average shortest paths and clustering coefficient, to quantitatively analyze the network structure characteristics of PFN in China in five stages.

Network density (ND) refers to the degree of the closeness of connections between node cities in PFN, the higher the value of ND, the more tightly the cities are connected. The calculation formula is the following:

(1) $$\mathrm{ND}=\sum_{i=1}^{n}\sum_{j=1}^{n}w_{ij}/n\left(n-1\right)$$

where ND is the network density, *i* and *j* are any two node cities, *n* is the number of node cities in PFN, and *w_ij_* is the strength of the relationship between the two cities, which is represented by BMI in this paper.

Average path length (PL) distinguishes an easily negotiable network from one which is complicated and inefficient, with a shorter average path length being more desirable [31]. In a real network like PFN, a short average path length facilitates the quick transfer of population and reduces costs. In general, PFN have a very short average path length leading to the concept of a small world network (SWN) where every city is connected to everyone else through a very short path. The calculation formula is as follows:

(2) $$\mathrm{PL}=\frac{2}{n\left(n+1\right)}\cdot \sum_{i,j}^{}d_{ij}$$

where *n* is the number of vertices in PFN. let *d_ij_* denote the shortest distance between *i* and *j*, assume that *d_ij_* = 0 if *i* = *j*, or *j* cannot be reached from *i*. 

In network theory, the clustering coefficient is the coefficient used to describe the degree of clustering between the notes of a network. Specifically, it is the degree to which the adjacent points of a point are connected to each other. The definition of the total agglomeration coefficient is based on the closed three-point group (adjacent three-point group). The average agglomeration coefficient (C) is specifically the arithmetic average of the local agglomeration coefficients of all vertices [31]. The calculation formula is as follows:

(3) $$C=\frac{1}{n}\sum_{i=1}^{n}\frac{2\delta_{G}\left(i\right)}{k_{i}\left(k_{i}-1\right)}$$

where *n* is the number of vertices in PFN. let $\delta_{G}\left(i\right)$ denote the number of subgraphs with 3 edges and 3 nodes of *G*, for a directed graph, *k_i_* is the number of adjacent nodes of vertex *i*.

#### 2.2.3. Effective Distance

There is no doubt that long distances can block the spread of disease; however, Brockman et al. (2013) pointed out that geographical distance is not the decisive factor through the empirical study of SARS in 2003, and put forward the concept of “effective distance” [23]. In PFN, we redefine the effective distance between city A and city B as the length of the most likely route for a passenger which is treated as a random walk particle. It will randomly visit the surrounding cities according to the traffic flow of the two cities. Therefore, the most likely route is the path with the highest probability which is converted from traffic flow. In effective distance model, the mobility matrix P is constructed based on the population flow between two cities (represented by BMI). Given the flux–fraction 0 ≤ *p_mn_* ≤ 1, i.e., we define the effective distance *d_nm_* as:

*d_mn_* = (1 − log *p_mn_*) ≥ 1 (4)

where *p_mn_* is the fraction of travelers that from node n to node m, it reflects the idea that a small fraction of traffic is effectively equivalent to a long distance. Realistic, the flow of population is generally asymmetric, so is the effective distance, i.e., *d_mn_* ≠ *d_nm_.*

## 3. Evolution Pattern of PFN

### 3.1. Changes in the Scale of Population Mobility

**Figure 2:**
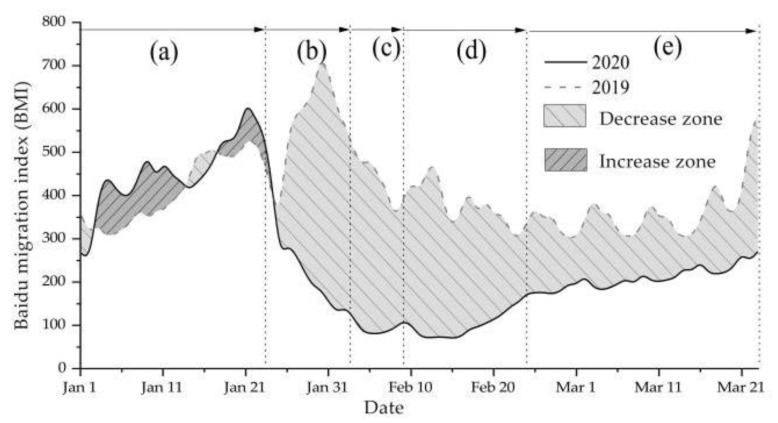
Comparison of trends of population mobility in China in 2020 and 2019. In order to facilitate comparative study, we convert the two sets of data in a unified way according to the lunar calendar, correspondingly, the data for 2019 are from 12 January 2019 to 4 April 2019. (**a**–**e**) represent five stages, which we distinguished earlier.

The overall scale of national migration in 2020 decreased by 36.85% compared with the same period last year (Figure 2). With 24 January as the demarcation point, the scale of national population migration changed from the steady growth trend of stage 1 (1 January–23 January) to the cliff decline of the epidemic outbreak period (stage 2). There is no peak of population migration back to the city at this stage, and the migration index is only 38.72% of that in the same period in 2019. After China entering stage 3 (3 February–9 February), the grim situation of epidemic prevention and control in the whole country after the festival has not subsided. The plan for enterprises’ resumption and production has been suspended and postponed many times by the government. Additionally, strict traffic control measures are still being implemented nationwide, and the “pause key” of population movement between cities has been pressed. During this period, the flow intensity decreased by as much as 78.52%. The number of population migration increased significantly after 16 February, and the low trend of population mobility hovered for a week before it gradually increased, and entered the period of resumption of work and production in some areas (10 February–25 February). Due to the risk of infection, local governments and enterprises decided to postpone the plan to resume work on February 10. However, sustained and normal operation of society needs to restore the vitality of the market and society. Industries which are essential to people’s daily life (such as water supply, gas supply, power supply, communications, supermarkets, farm produce market, etc.) and industries necessary for epidemic prevention and control have entered the stage of resumption of work and production since February 16. At this stage, the intensity of population mobility gradually rebounded, but the decline was still as high as 70.54% compared with the same period in 2019. Since March, the national population mobility entered an orderly recovery period, but the rate of decline was still as high as 40.51% compared with the same period in 2019.

### 3.2. Spatial Pattern Changes of PFN

**Figure 3:**
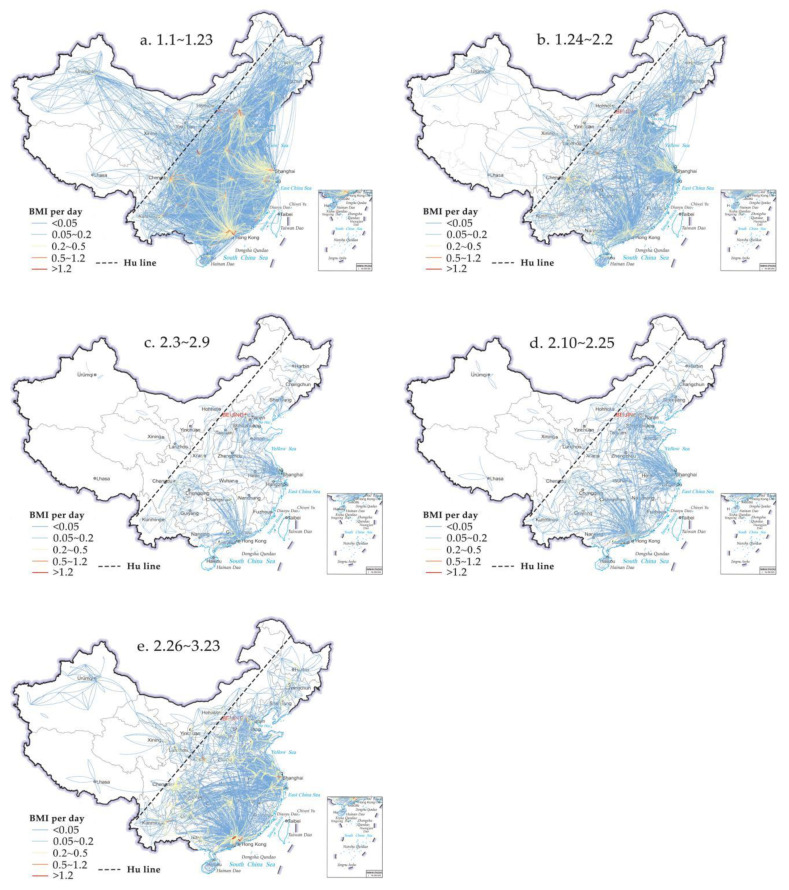
Spatial pattern evolution of PFN in China. Note: due to the asymmetric direction of the villages in PFN, there are two arcs between every two nodes. We set clockwise as the direction of population flow. Baidu Qianxi provide “Baidu migration index (BMI)” to indicate the size of inter-city migration, we convert BMI into the number of population movements using the actual number of inter-city/within-city population flows, provided by official WeChat accounts of Baidu Qianxi. When doing OD network analysis, we need to delete some unimportant links to intuitively and clearly present the information in this figure, so we take BMI equal to 0.003, that is, the actual migrant population is 100 (the rounded data) as the research threshold (**a**–**e**).

By comparing and analyzing the PFN in five periods (Figure 3). We found that the scale of urban population flow in China has shown a process of rapid reduction and then a slow recovery, and obvious hierarchical characteristics are presented in all phases: a rhombic structure pattern with the four urban agglomerations of Pearl River Delta, Yangtze River Delta, Beijing–Tianjin–Hebei region, Sichuan and Chongqing Urban Agglomeration has not changed significantly. Hu Huanyong line, the important dividing line of population geography in China, also dominates the spatial distribution of China’s PFN. more than 90% of the network connections are located in the southeast of Hu line, especially in the third stage, this value is as high as 94.86%. The vast region western of Hu line only formed scattered central nodes represented by Urumqi, Lanzhou, Xining, Hohhot and other provincial capitals. Bounded by Hu Huanyong Line (abbreviation: Hu line), the spatial differentiation of PFN presents a pattern of high in the southeast and low in the northwest. Meanwhile, during the Spring Festival travel rush (stage 1), Wuhan, as the largest transportation hub in central China, connects many cities including the four major urban agglomerations, it behaves as a regional core node in PFN. After the outbreak of COVID-19, Wuhan and other cities in Hubei province were the first to implement the lockdown through traffic control measures. The cross-city travel of residents in the province is strictly restricted. The “central downfall” pattern of population flow in China has been formed (See Figure 3b–e).

Figure 3 clearly shows that there is a close relationship between the scope and scale of population flow and the city level. The high-value network connections appear in the populous municipalities, provincial capitals and large urban agglomerations. As the epidemic grows worse, the scope and scale of population movements have been greatly reduced. However, first-tier cities continue to be hubs of population movement, such as Beijing, Shanghai, Guangzhou, Shenzhen, etc. Therefore, the hierarchical structure dominated by core nodes is an important feature of PFN. Furthermore, we can easily find that the geographical proximity effect is another important migration pattern, which is particularly obvious in the several stages after the outbreak of COVID-19. In order to reduce the impact of the epidemic and block the spread of the epidemic, each province has implemented different levels of traffic control measures in China, the short-distance migration (including the population flow between the cities in the same province and the neighboring provinces) replaces the long-distance cross-province migration. Since entering the stage of nationwide strict control (stage 3), the number of OD lines (Origin-Destination movement lines) in the same province has surged to 57. 58% from 30.53% (Table 1, stage 1). It was not until March, the proportion of interprovincial OD lines is gradually rising back to similar levels as in the pre-holiday spring period (stage 1). Yet, according to the volume of population flow, the proportion of migration in the same province increased from 49.94% (stage 1) to 66.03% (stage 3), and then fluctuated slightly, but basically maintained at the same level of about 66%. It has not fallen back, even increased slightly (66.86%) into the orderly recovery period in March (stage 5). In addition, we can also see that the epidemic does inhibit the radius of migration for people to travel from the changes in migration distances. The average migration distance was reduced by 49.87% before and after the COVID-19 outbreak, although it increased to 454.33km during the orderly recovery period (stage 5). In terms of weighted migration distance (take the traffic flow of each OD line as the weight) short-distance migration is still the main pattern for Chinese returning to the city in the later period of the epidemic, the average weighted migration distance is always maintained at about 250 km in this stage.

Hubei Province, as the hardest hit area of COVID-19, took the lead in implementing strict traffic control measures, which may lead to some new characteristics of population flow: lower scale of migration and higher proportion of intra-provincial migration (Table 2). Before the Spring Festival, the percentage of immigration and emigration flows of Hubei province are roughly in line with its population size as a percentage of the country (around 4%). However, it rapidly reduces to 1.28% and 1.37% after outbreak, and showing a slight oscillating trend. In which the proportion of people moving into Hubei province continued to drop to 1.04%. Until 23 March, Hubei’s public health emergency response level is still at level 1 due to the risk of an outbreak rebound, and transportation is still subject to strong control restrictions. The population flow is mainly concentrated between cities within the province, where the proportion of people moving in and out of the country remains at a high level of around 80%. Surprisingly, the proportion of immigrants from other cities in the province reached 92.27% during the stage 5, which is related to the latest lifting of traffic control restrictions in Hubei Province. Meantime, there was a small amount of short-distance cross-province movement between Hubei and its neighboring provinces, such as the migration between Enshi ↔Chongqing, Xiangyang↔ Nanyang (Henan Province), Jingzhou ↔ Changde (Hunan Province), Shiyan ↔ Ankang (Shaanxi Province), etc. The migration distance of these OD lines is generally less than 250 km.

### 3.3. Network Topology Analysis

The network density of PFN for the first stage (1.1–1.23) is 0.188 (Table 3), which is the highest of all stages, and the network density gradually decreases to 0.065 in the third stage in which traffic control is the tightest, and the flow of population is greatly restricted. We can also see the trend from the number of lines of PFN, from the first stage to the third stage, the number of network edges is reduced to about 1/3, which shows that the PFN becoming decentralized. After that, the network density showed a V-shaped recovery. Until the fifth stage, network density increased to 0.117, which has not returned to the level of the first stage, and it can still be shown that there was increased mobility and overall accessibility between cities in China.

Similar trends are reflected in the clustering coefficient and the average path length. Table 1 shows that the average network clustering coefficient for the period from 1 January–23 January is 0.74, which is larger than other stages. Correspondingly, the average path length is the shortest (1.881) in the first stage. After the outbreak of COVID-19, traffic flow was controlled, PFN has changed accordingly. While the clustering coefficient is decreasing, the average path length is increasing. In the third stage, the clustering coefficient reduced to the lowest value (0.632), meanwhile, the average path length arrived at the highest value (2.546). After that, with the resumption of work and production of enterprises, the flow of population between cities has become increasingly active. In the fifth stage, the average clustering coefficient has increased to 0.678, and the average path length decreased to 2.295, which demonstrates an increase in network connectivity and the development of a tighter overall network. However, there is still a significant gap from the first stage. 

In summary, COVID-19 has had a tremendous impact on the network topology of PFN in China. Only the first stage PFN has a large clustering coefficient and relatively short average path length, which is more consistent with the characteristics of a small-world network. In other phases, the movement of people was greatly restricted by strict traffic control during the epidemic. The level of network connection between city nodes is lower than that of the first stage. The spatial distribution of network connections tends to be more centrally distributed. This can also explain why the distribution of population movement in the four phases of the outbreak in Figure 3 above is more concentrated in the Yangtze River Delta, Pearl River Delta, Beijing–Tianjin–Hebei region and Chengdu-Chongqing Urban Agglomeration. Its spatial heterogeneity is greater. Relatively speaking, it is slightly less networked than the stage of returning home before the festival.

## 4. An Explanation of Correlation between PFN and COVID-19: Effective Distance

**Figure 4:**
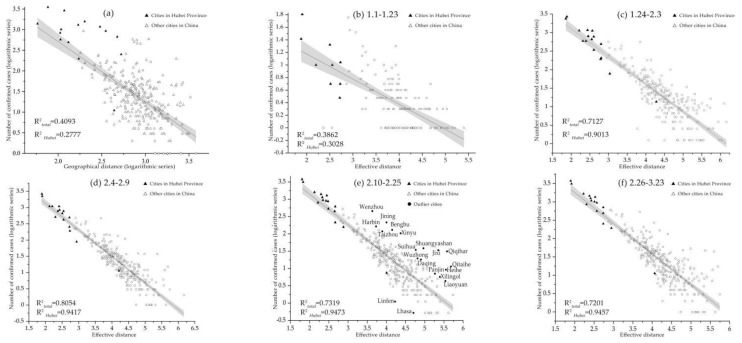
Factors correlated with confirmed COVID-19 cases. The relationship between the log-transformed geographical distance from Wuhan to nearly 300 cities and the log-transformed number of confirmed cases (**a**); (**b**–**f**), relationship over time between the log-transformed number of confirmed cases and the effective distance from Wuhan to destination cities.

Under the hypothetical precondition that the regional distribution of the epidemic is dominated by the spread in Wuhan, the migration of people from Wuhan to other places plays an important role in determining the geographical location of confirmed cases of COVID-19 nationwide. It is well known that distance is an important geographical factor in restraining population migration. We analyzed the relationship between the cumulative number of confirmed cases (as of 23 March, 24:00) and the geographical distance from Wuhan to nearly 300 cities. The results showed that the number of diagnosed patients decreased with the increase of distance, but the correlation coefficient R^2^ is 0.4093 (Figure 4a). The low negative correlation indicates that the direct role of space distance in virus transmission is overestimated. Therefore, we introduce the effective distance model proposed by Brockmann and Helbing (2013) to fit the correlation between distance and confirmed cases in stages. Indeed, we find a strong correlation between total population flow and the number of infections in each city. It was found that there was a significant fitting relationship of more than 0.7 in other periods except that the fitting coefficient of the first stage was slightly worse (R^2^ = 0.3774). The fitting determination coefficient of the strict control stage is as high as 0.8054, compared with it, the cities in Hubei province have a higher goodness of fit, the correlation coefficients R^2^ for the last four stages are all more than 90%. This shows that the effective distance produces a higher criticality than the spatial distance (Figure 4d). In other words, with the progress of transportation technology, the propagation path of the epidemic is no longer determined by the traditional sense, but along the most likely path with traffic characteristics. 

Compared with the fitting situation of the five stages, it is found that the significant level (R^2^) shows the trend of inverted “V” shape which rises first and then decrease (Table 4). This may be closely related to the immature and effective early detection ability and virus’s long incubation period [32]. In the stage of stick control, the traffic control measures between cities greatly blocked the spread of the epidemic source, and the R^2^ reached the maximum value 0. 8054 (R^2^ = 0.9473, cities in Hubei Province). It is worth noting that although the effective distance can explain most of the variation of epidemic scale, there are often exceptions in reality due to its complexity, such as Wenzhou, Taizhou, Jining and Xinyu. After that, with the gradual resumption work in non-key epidemic areas, population mobility, especially the import of overseas population, has become increasingly active, which undoubtedly increases the risk of epidemic transmission. Among them, the northeastern border cities of Qiqihar, Jixi, Qitaihe and Heihe have become high-risk areas for the importation and spread of cases from abroad (Figure 4e). This lowers the coefficient of determination for the fit of the effective distance model R^2^, but it still reaches 0. 7201. Thus, effective distance based on population movement data has a better explanation for the probability of spatial spread of the epidemic.

## 5. Discussion

The existing related research mainly focuses on the deduction and verification based on the SIR, SIRE classical infectious disease spreading prediction model, but the spreading factors of the large-scale epidemic situation are extremely complex. It is closely related to the physical condition, social intensity, local medical level and even the natural environment of the susceptible population [24], which makes the final prediction prone to larger errors. From a relatively macroscopic research perspective, the study abstracts a city as a network node and replaces the conventional geographic distance with the effective distance of population migration probability orientation. Additionally, through the comparative study of spatial distance and effective distance models at two scales for cities in Hubei province and nationwide, we find that the coefficient of determination of the effective distance model are at a high level in many periods, which also indicates that the method has good stability. It can be inferred that inter-city traffic flow is the key factor that determines the scale and rate of virus transmission between cities, which is confirmed once again by the late spread of the epidemic on a global scale. In the current context of globalization, geographical distance is no longer the most important factor in the spread of an epidemic. Meanwhile, with the advent of the era of information and communication technology, the wide use of big data and open data provides new methods and approaches for urban research, and also provides the possibility for real-time prevention and control of the epidemic. In addition, the registered residence system should be adjusted gradually, and the migrant population should be allowed to become a new city citizen as soon as possible, so as to gradually reduce the large-scale “pendulum” movement.

It is undeniable that there may be some shortcomings in the analysis of population flow patterns and its correlation with epidemic transmission based on Baidu LBS migration data. The shortcomings are mainly as follows: (1) Due to the limitations of generating and acquiring geographical behavior big data [16] and the attributes of mobile smart terminal users, the objects of location acquisition are biased in the analysis of inter-city population migration, and most of the objects’ travel routes may be disassembled, making it impossible to determine the users’ complete travel routes. In order to protect users’ privacy and so on, the data does not display the social attributes (occupation, gender, age) of the migrant population, and it is impossible to know the purpose of migration and duration of stay. In addition, Baidu Migration only shows the migration amount proportion of the top 50 or 100 destination cities in each day, and cities with less than 80% of the total migration amount account for 29.43%, which does not cover all the destination cities, so using its migration probability to calculate the effective distance will inevitably lead to some deviations, and the goodness of fit is slightly lower than the results of the study by Jia et al. using mobile phone signaling data [24]. (2) As the COVID-19 epidemic was unique in its suddenness, rapid rate of spread, widespread coverage, and coincided with Chinese New Year, factors such as the early detection capacity and scale of detection, changes in statistical calibers, and differences in the level of medical care among cities may cause large errors in the release of statistics on the number of confirmed cases. According to the better fitting result of the later effective distance model, it can be inferred that in the early stage of the epidemic development, other cities in Hubei Province, which are closer in effective distance, should have more serious epidemic in theory. However, there were no confirmed cases in Xianning, Xiangyang and Huangshi in the notification of the epidemic on 24 January, which led to the decision coefficient R^2^ of cities in Hubei Province in the first phase even being lower than the national level. (3) Although the effective distance model can estimate the spread track and scale of epidemic situation at a high fitting level, it cannot improve the interpretation degree of the model by using only the effective distance single variable. It is suggested that more variables should be included in the analysis of epidemic scale and the study of early warning in order to achieve better modeling effect.

## 6. Conclusions

Based on Baidu LBS big data and social network methods, this study attempts to explore the spatial pattern and evolutionary characteristics of urban population flow network structure under the influence of the COVID-19 in China and adopt the effective distance model to further explore the correlation between population flow and the spread of the epidemic to obtain the better fitting effect of the model. The results show that COVID-19 epidemic has had a tremendous impact on the scale and direction of population flow in urban areas across the country, resulting in a “central collapse” pattern in some areas, but it has still not broken the overall pattern of “eastern is more than western in the mass” of population distribution and migration on both sides of the “Hu Line”. The geographical proximity effect and the hierarchical structure dominated by the four urban agglomerations of Yangtze River Delta, Pearl River Delta, Beijing–Tianjin–Hebei region and Chengdu-Chongqing Urban Agglomeration, were the two major patterns of population movement during the epidemic period. The population flow during the epidemic prevention and control period is mainly intra-provincial mobility, accompanied by a small number of high-intensity short-distance cross-provincial travel. The long-distance cross-provincial migration has decreased, especially in Hubei Province, which is the worst affected area of the epidemic. Through the analysis of network structure, it is found that only the first stage of population flow network has a large clustering coefficient and a relatively short average path length, which is consistent with the characteristics of small-world network. The impact of the epidemic significantly altered the topological characteristics of the population flow networks in the remaining phases. Based on the regression analysis of two kinds of distance models, the results show that there is a significant linear relationship between the effective distance from Wuhan to the destination and the cumulative confirmed case series of the COVID-19, both at provincial level and national level.

## Figures and Tables

**Table 1:** Migration distance and proportion of migration within the province of PFN.

Statistical Indicators	1.1–1.23	1.24–2.2	2.3–2.9	2.10–2.25	2.26–3.23
	Stage 1	Stage 2	Stage 3	Stage 4	Stage 5
Total number of OD lines (BMI > 0.003)	10472	7002	2466	3813	6579
Average migratory distance (km)	615.58	470.71	308.61	373.17	454.33
Weighted migratory distance (km)	363.13	270.32	246.64	251.88	251.24
Proportion of OD lines within provinces	30.53%	40.95%	57.58%	48.59%	39.05%
Proportion of flow within provinces	49.94%	66.03%	66.52%	65.07%	66.86%

**Table 2:** Statistical table of PFN in Hubei Province.

Statistical Indicators	1.1–1.23	1.24–2.2	2.3–2.9	2.10–2.25	2.26–3.23
	Stage 1	Stage 2	Stage 3	Stage 4	Stage 5
Emigration from Hubei/Total emigration	3.9%	1.37%	1.62%	1.71%	1.36%
Immigration to Hubei/Total immigration	4.34%	1.28%	1.63%	1.72%	1.04%
Percentage of emigration from Hubei Province	78.71%	74.29%	85.22%	84.96%	80.89%
Percentage of immigration to Hubei Province	61.47%	79.71%	84.90%	85.03%	92.27%

**Table 3:** Network indicator of PFN in different periods.

Network Indicators	1.1–1.23	1.24–2.2	2.3–2.9	2.10–2.25	2.26–3.23
	Stage 1	Stage 2	Stage 3	Stage 4	Stage 5
Network density	0.188	0.125	0.065	0.101	0.117
Average clustering coefficient	0.74	0.698	0.632	0.639	0.678
Average path length	1.881	2.171	2.546	2.36	2.295
Total OD lines	17,508	13,485	6840	10,480	12,630

**Table 4:** The results of linear regression analysis.

Model	Spatial Scale	Phases	95%CI	*t*-Value	Prob > F	R-Square
Geographical distance	National China	1.1–3.23	−1.464 (±0.101)	−14.445	0.000	0.4093
	Hubei Province	1.1–3.23	−1.049 (±0.452)	−2.320	0.036	0.2777
Effective distance	National China	1.1–1.23	−0.399 (±0.041)	−9.682	0.000	0.3862
		1.24–2.2	−0.802 (±0.030)	−26.305	0.000	0.7127
		2.3–2.9	−0.820 (±0.025)	−32.908	0.000	0.8054
		2.10–2.25	−0.824 (±0.029)	−27.749	0.000	0.7319
		2.26–3.23	−0.825 (±0.030)	−27.645	0.000	0.7201
	Hubei Province	1.1–1.23	−1.012 (±0.512)	−1.977	0.079	0.3028
		1.24–2.2	−1.060 (±0.100)	−10.561	0.000	0.9013
		2.3–2.9	−1.137 (±0.093)	−12.231	0.000	0.9417
		2.10–2.25	−1.147 (±0.072)	−15.862	0.000	0.9473
		2.26–3.23	1.151 (±0.074)	−15.615	0.000	0.9457

## Data Availability

The data presented in this study are available on request from the corresponding author.
